# Dispersive Micro-Solid Phase Extraction for Sensitive Determination of Methotrexate from Human Saliva Followed by Spectrophotometric Method

**DOI:** 10.31557/APJCP.2020.21.6.1531

**Published:** 2020-06

**Authors:** Fatemeh Soghra Jahed, Samin Hamidi, Mohammad Galehassadi

**Affiliations:** 1 *Food and Drug Safety Research Center, Tabriz University of Medical Science, Tabriz, Iran. *; 2 *Department of Organic Chemistry, Azarbaijan Shahid Madani University, Tabriz, Iran. *

**Keywords:** Methotrexate, saliva, dispersive micro solid phase extraction, spectrophotometry

## Abstract

For biological assessing of hospital personnel occupationally exposed to antineoplastic drugs, highly sensitive and accurate methods are required. Methotrexate (MTX) is an anticancer agent that is widely used in a variety of human cancers. For the first time, dispersive-micro solid phase extraction (D-µ-SPE) has been applied for determination of low levels of MTX in saliva samples. The method is based on rapid extraction of MTX using graphene oxide adsorbent. The sample preparation time is decreased by the fact that the adsorbent dispersed in the sample solution and extraction equilibrium can be reached very fast. This significant feature which obtained with this method is of key interest for routine trace laboratory analysis. The influence of different variables on D-µ-SPE was investigated. Under optimum conditions, the calibration graph was linear over the range of 10–1,000 ng/ml. The relative standard deviations are better than 9.0%. The proposed method was successfully applied for the determination of MTX in patient samples.

## Introduction

**Figure 1:**
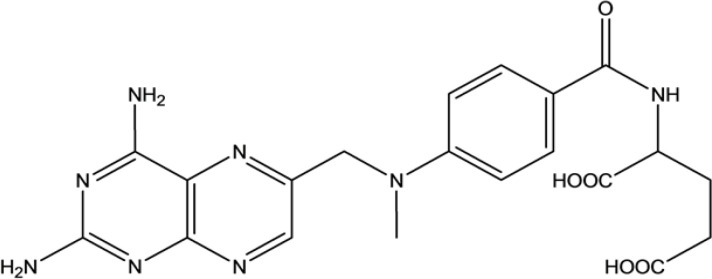
Chemical Structure of Methotrexate

Methotrexate (Figure 1) is an anticancer drug belonging to a class of chemotherapy drugs. It has the potential to be used as a treatment for cancers including breast cancer, lung cancer, leukemia, lymphoma, osteosarcoma and also used in the treatment of some autoimmune diseases. MTX mechanism of action in cancer therapy is performed by inhibiting cellular enzymes and preventing cell replication. It is generally administered orally and after absorption, most of the drug is excreted intact in urine.

Determination of MTX in biological samples is needed for therapeutic drug monitoring (TDM), pharmacokinetic studies, bioequivalence tests, toxicology, and forensic science (Fleisher, 1993; Flores et al., 2005). In cancer therapy, high-dose of MTX is used that have serious toxic effects. MTX has many serious adverse effects, such as myelosuppression. To minimize toxicity, safe and effective MTX therapy, monitoring of biological MTX level is very important to improve the safety of medicinal therapy (Emara et al., 1998; Li et al., 2015; Asadian et al., 2017). Several methods have been reported to determine the MTX in different biological matrices using high-performance liquid chromatography (HPLC) (Emara et al., 1998; Hroch et al., 2009; Moghbel et al., 2010; Uchiyama et al., 2012; Begas et al., 2013; Li et al., 2015), electrochemical impedance spectroscopy and cyclic voltammetry (Wei et al., 2014; Asadian et al., 2017), fluorescence (Nelson et al., 1977; Chen and Zhang, 2008; Jouyban et al., 2011), surface-enhanced Raman scattering (SERS) (Yang et al., 2014) and ultra performance liquid chromatography-electrospray ionization-tandem mass spectrometry (UPLC-ESI-MS/MS) analysis (Fabrizi et al., 2016). In the recent years less common or unconventional matrices have emerging more significant owing to their invasiveness over the conventional specimens for example when taking blood from children and neonates is difficult (Gallardo and Queiroz, 2008; Hamidi et al., 2016; Hamidi and Alipour-Ghorbani, 2017; Hamidi et al., 2017a; Hamidi et al., 2017b; Jouyban et al., 2017). Effectiveness of saliva was assessed as a non-invasive and alternative specimen to urine and blood in terms of ease of collection, matrix simplicity, and drug detection periods. Most of the analytical methods reported for MTX are tedious, time-consuming and need highly skilled operators. UV-Vis spectroscopy is one of the inexpensive, low cost and simplest analytical tools that routinely used in almost all of the biomedical laboratories (Ghasemi and Niazi, 2005; Samari et al., 2010; Hamidi, 2018). The most frequent detection system employed for the determination of MTX is UV-Vis (Rubino, 2001). MTX is a strongly UV-Vis absorbing substance, due to the presence of the heteroaromatic chromophore. To overcome the difficulty of poor sensitivity in UV detectors, pre-concentration methods have been recommended; the most important step in the determination of an analyte is sample preparation (Vas and Vekey, 2004; Hamidi and Jouyban, 2015a; Hamidi and Jouyban, 2015b; Hamidi et al., 2015). 

Biological matrices more or less contain proteins, salts, and lipids which need to be treated prior to being coupled with the majority of analytical devices. A bioanalytical method has two major steps: sample preparation and sample quantification. Sample preparation is a crucial step and aims to simplify matrix and/or enrich analyte. Some estimates show that over 80% of an analysis process is dedicated to sample pre-treatment in order to introduce matrix-free extract into a measurement device. The ultimate aim of an extraction method is to provide the target analyte in solution with an appropriate concentration for quantification and to let it free from interferences that might exist in the original source of sample. Liquid-liquid extraction (LLE) and solid phase extraction (SPE) are the most popular sample preparation processes. Generally, LLE is performed with water-immiscible solvents to recover the analyte from the aqueous solution. However, LLE technique should be performed under conditions which provide a distinct and clean separation boundary between the extraction phase and the sample solution. Formation of rather stable emulsions is often a formidable obstacle during the phase separation, threatening efficient extraction performance, particularly when dealing with biological samples (Ruiz-Gutiérrez and Pérez-Camino, 2000; Poole, 2003).

Nowadays SPE is known as a general technique in sample pre-treatment. In conventional SPE, solid particles are embedded on a solid phase such as cartridge, fiber, and disk. The sample passed through the column by applying external pressure and they are usually recommended for single use (Ashri and Abdel-Rehim, 2011; Hamidi et al., 2018; Jafari and Hamidi, 2018). A sufficiently low flow-rate through the column of a specific sorbent is of prime importance in SPE. Residual solvent and sample molecules removal from the sorbents is very important in order to improve the reliability of the procedure. Another limitation of suffering in conventional SPE, in particular when dealing with complex matrices is clogging the sorbent pores which, in turn, affect the analyte retention time and sorbent capacity. Dispersive micro solid phase extraction (D-µ-SPE) is a new, rapid, simple and effective type of SPE, used for pre-concentration of trace amounts of target analytes (Jouyban and Hamidi, 2017). In this technique, adsorbent was added into the sample solution and separation process achieved only by centrifugation or an external magnetic field (Jouyban and Hamidi, 2017; Khezeli and Daneshfar, 2017). Graphene and Graphene-based materials are good candidates for sample preparation as in many studies have been directly used as an adsorbent in the D-µ-SPE process due to their unique properties e.g., having a high surface area to weight (2630 m2g-1), thermal and chemical stability and simple synthesis process (Ye and Shi, 2015). Graphene oxide (GO) is a functionalized form of graphene with different epoxy, hydroxyl, carbonyl and carboxyl surface groups (Ayazi, 2017). It is easily prepared from natural graphite by Hummer’s method (Shahriary and Athawale, 2014). GO with polar surface functional groups and delocalized π-electron system is an appropriate adsorbent for extraction of polar and aromatic organic compounds. 

In this work, GO was synthesized by the simple Hummer’s method and used for extraction of MTX from real human saliva samples. UV spectroscopy was used for determination of the analyte concentration. The developed method was validated according to FDA guidance and applied for the determination of MTX levels in two patients with acute lymphoblastic leukemia. 

## Materials and Methods

*Experimental*

*Materials and instrument*

MTX powder was purchased from Sigma (Sigma, USA). Acetonitrile (ACN), methanol, ethanol and acetone were obtained from Scharlau (Barcelona, Spain). Sodium hydroxide (NaOH), expanded graphite powder, sulforic acid (H2SO4, 98 %), hydrogen peroxide (H2O2, 30 %), KMnO4, hydrochloric acid (HCl) were purchased from Merck (Darmstadt, Germany). 

The UV-Vis spectrophotometer (Thermo, USA) accompanied with Thermo Insight software was used to determine the concentration of MTX. A 350 µl quartz cell was used to record spectrums and absorbance measurements. The absorbance was recorded at the wavelength of 375 nm. 

*Standard solutions and saliva samples*

Stock standard solution of MTX (1,000 µg/ml) was constructed in methanol and stored in -4^o^C. Drug-free saliva samples were provided by volunteer donors and transferred in polypropylene microtubes and frozen. Saliva samples thawed at room temperature unassisted before daily experiments. Daily standard MTX solutions were prepared in drug-free saliva sample and diluted with the same matrix. The required calibrator solutions (0.01, 0.05, 0.1, 0.5 and 1 µg/ml) were prepared in the same manner. Two samples were obtained from patients receiving MTX who had signed consent forms approved by the ethics committee, Tabriz University of Medical Sciences. These Samples were also collected in polypropylene tubes and stored in refrigerator until analysis.

*Synthesis of GO*

GO was prepared and characterized by the method mentioned in our previously work (Jouyban and Hamidi, 2017). In brief, graphite (2 g) and sulfuric acid (50 ml) was purred in 1,000 ml flask and stirred at 0-5^o^C in the ice bath for 2 h, then potassium permanganate (6 g) slowly added to the mixture. In this step the reaction temperature should be controlled and keep lower than 15^o^C. After that, the reaction mixture stirred at 35^o^C until pasty brownish color obtained and continuously stirred at this temperature. After 30 min the mixture diluted by addition of 100 ml deionized water, the temperature rapidly reached to 98^o^C and color changed to brown. Dilution was continued by adding 200 ml deionized water. The reaction terminates by adding 10 ml H_2_O_2_ and yellow color was appeared. After filtration, the obtained GO washed several times with 10% HCl and deionized water and dried at 70^o^C for 24 h.

*Dispersive micro-solid phase extraction (D-µ-SPE) procedure*

The D-µ-SPE performance was done as follows: First 0.5 ml of the saliva sample was put in a 15 ml tube spiked with MTX and diluted six times with deionized water. Then, the pH of this solution adjusted to 4 with 30 µl of HCl 0.1 M. Next, 3 mg GO weighed and dispersed into the mixture. The tube was placed in an ultrasonic bath for 6 min, then the adsorption procedure was completed by vortexing for 1 min to accelerate the mass transfer of analytes through increasing the interfacial area between the solid sorbent and sample solution. The MTX-loaded GO (MTX/GO) centrifuged in 6,000 rpm for 1 min and the supernatant was filtered off. Afterward, 700 µl of acetone as desorption solvent was added to the sedimented MTX/GO, this mixture sonicated for 3 min, then filtered by centrifuge 6,000 rpm for 1 min. The supernatant was kept for analyzing the drug content. The acetone was evaporated by nitrogen gas, and the residue was dissolved in 200 µl ACN. Finally, the amount of MTX was determined by UV spectrophotometer at 375 nm. 

## Results

In order to improve the extraction capability, a set of effective parameters were studied including sample volume, pH of sample solution, amount of adsorbent, adsorption and desorption time and type of eluting solution. These preliminary studies were performed in human saliva samples. Extraction of MTX performed through π-π interaction between aromatic part on drug structure and delocalized π-electron system of GO surface, also the formation of hydrogen bonding of carboxyl and amine group of MTX with carboxyl and hydroxyl groups on the surface of GO is considered. 

*Sample volume*

For the analysis of a real sample using pre-concentration, the sample volume is one of the most important parameters for obtaining high enrichment factor. To do this, 0.5 ml of the saliva sample after MTX spiking was used in all studied volumes. Volumes of sample solutions were changed from 1.0 to 10.0 ml in test tubes. The highest analytical response was achieved at the sample volume of 3.0 ml. Therefore, 3.0 ml was chosen as the optimum sample volume. 

*pH optimization*

**Figure 2:**
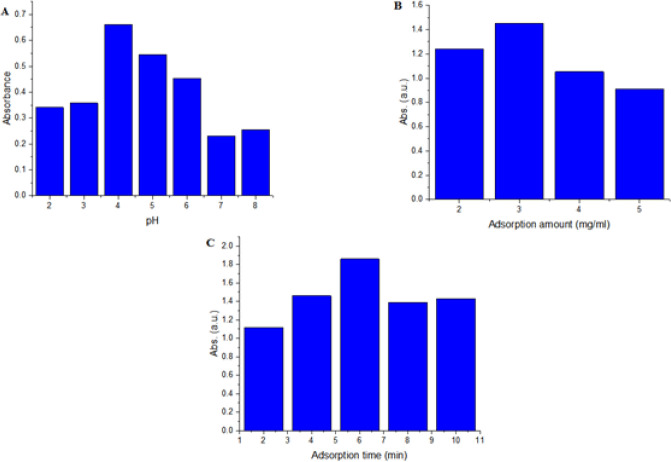
Effect of pH (A) Various Amounts of GO Sorbent (mg/mL) (B) and Contact Time (C) on the Extraction Efficiency

The pH values were investigated between 3.0 and 11.0 by the addition of 0.1 M HCl or 0.1 M NaOH solutions. From Figure 2A, it was revealed that the extraction has been occurred best in acidic condition. The MTX has three different pKa values, 3.8, 4.8 and 5.6 due to the different chemical sites. In pH= 4, the MTX is mostly in undissociated form, thus the solubility is reduced and there upon maximum extraction is reached. It can be concluded that the electrostatic interactions between MTX and adsorbent showed a moderate role in the adsorption process. Since the pH of the real saliva samples was generally in the range of 6.5–7.0, there it is needed to adjust the pH of the sample solution before the adsorption process. 

*Amount of GO sorbent*

The optimum amount of the adsorbent for maximum take up was determined in the range of 2–5 mg/ml. According to Figure 2B, 3 mg/ml of the adsobent was enough to pre-concentrate the MTX, at the studied concentrations. 

*Adsorption time*

In this experiment, adsorption time means the time interval from the beginning of the injection and its end just before using an external magnetic field. The time of adsorption is a necessary parameter to ensure the sufficient contact between sorbent and analyte. In the present stage the effect of time on extraction efficiency was checked in different contact times; 2, 4, 6, 8 and 10 min. As shown in Figure 2C, the equilibrium of the extraction process was reached at 6 min so recovery increased in this time. Such a fast equilibrium between the adsorbent and analyte is reasonable due to the high hydrophilicity of adsorbent and its large accessible surface for extraction. With extending the time over 6 min, no significant change in analytical response was observed and subsequent experiments were set at 6 min. This observation could be as the result of nanoparticle agglomeration upper than the equilibrium point then reduction of surface area for drug adsorption.

*Desorption conditions*

**Figure 3:**
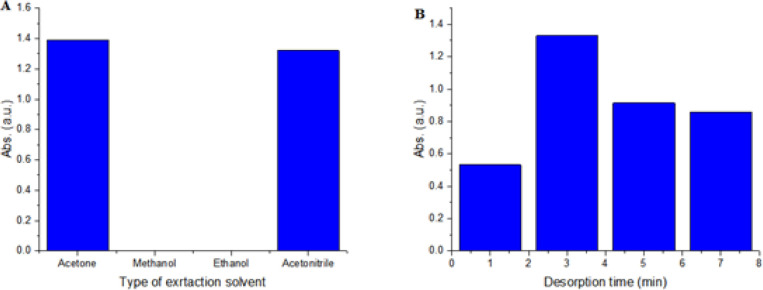
Effect of Type of Elution Solvent (A) and desorption solvent volume (B) on the extraction efficiency of the GO adsorbent

In order to choose the best eluent, MTX was desorbed with various eluting solutions such as ACN, acetone, methanol, and ethanol. As shown in Figure 3A, the desorption capacity of acetone is much better than that other eluents. Acetone with highest extraction efficiency is selected as desorption solvent. 

In order to complete desorption of the analyte from GO, the effect of desorption time on desorption capacity was also evaluated. Desorption time (1, 3, 5, 7, and 9 min) was also investigated. As shown in Figure 3B, 3 min is sufficient to desorb the MTX.

*Method validation*

The required parameters for validation of an analytical method according to the US FDA guideline are linearity, lower limit of quantification (LLOQ), upper limit of quantification (ULOQ), precision, accuracy, recovery, sensitivity, selectivity, and robustness (Food, 2001). In present paper required aspects for an analytical method have been covered including; linearity, accuracy, precision, recovery, stability, and selectivity. 

*Linearity*

**Figure 4:**
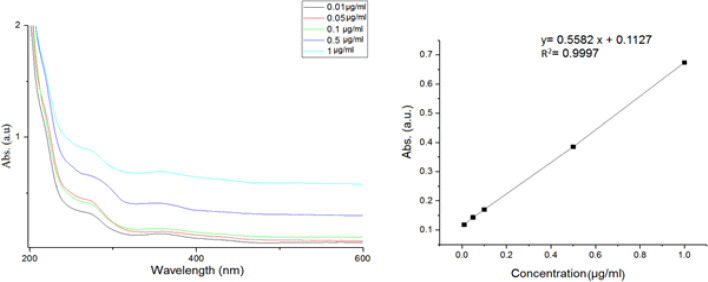
UV–vis Absorption Spectra of MTX at Increasing Known Concentrations

The calibration curve generated over the MTX concentration range of 0.01-1 µg/ml, with three replicates at each concentration level. For spiked saliva samples the correlation coefficient was greater than 0.999. Linear regression analysis of the results yielded an equation of y = 0.409 CMTX (µg/ml) + 0.142. Figure 4 shows the calibration curve related spectrum obtained by spectrophotometer in an optimized condition. 

*Precision, accuracy and recovery*

Precision was calculated in accordance with the FDA recommendation in two stages. Intra-day repeatability was assessed by analyzing five consecutive injections of MTX-spiked samples in three varying concentrations. The inter-day analysis was performed by quantification of the same concentrations on five consecutive days.

According to the FDA guideline the RSD% values lower than 20% for LLOQ and 15% for other occasions which are acceptable for bioanalysis. The accuracy of the method was defined as a relative error (RE%) calculated using the following equation:

$\mathrm{RE\%}=100\times (\frac{\mathrm{Found value}-\mathrm{Normal value}}{\mathrm{Normal value}})$

 Equation 1

The relative recovery (RR%) is defined as the measured concentration of the drug divided by its actual concentration. The intra-day and inter-day precisions, accuracy and recovery were performed by five replicates of 25, 50, and 100 ng/ml. The precision, accuracy, and recovery calculations are detailed in Table 1.

*Stability*

In this work, the stability tests covered the main concerns which target the stability of the components; when samples come to freezing as well as they come to stand at the bench. As the samples are severally frozen and thawed; thus, the effect of multi-freeze/thaw cycles should be concerned. The stability of the analyte should also be assessed in the sample preparation step until the analysis has been completed to figure out any degradation during the sample preparation. Table 2 shows the results of stability evaluation.

*Selectivity*

For spectrophotometric determination, it is necessary to investigate the acceptable levels of selectivity. Sample preparation is the first step to prone the sensitive and selective analytical method. Selectivity demonstrates the ability of the developed method to quantify the intended analyte among the compounds present in the sample. According to the FDA guideline, the drug-free saliva sample was collected from three different sources and applied into the spectrophotometer after applying D-µ-SPE. The blank samples showed no suspected interferences at absorbance area of the intended analyte and/or additional compounds did not have UV absorption at that wavelength. It means that routine constituents of extracted residual had no interferes with the analysis performance. So, the proposed method differentiates the target analyte in presence of potential interferences.

Selectivity of the method was ensured by adding 100 ng/ml of different probable co-administrated drugs (e.g., vancomycin, morphine, clonazepam, cetirizine, atorvastatin and cyclosporine) and most routinely administrated drugs such as acetaminophen and diazepam in saliva samples. The acceptable tolerance of accuracy should not exceeded 20% as was observed in the present study. Investigating of the interference effect of other drugs is presented in Table 3. As can be observed that the absorbance of investigated co-administrated drugs is not same as MTX. The obtained results confirmed that MTX could be successfully determined in real human saliva samples without the interference of a variety of routine compounds such as proteins, salts and usual co-administrated substances.

**Table 1:** Assay Precision, Accuracy and Recovery of Saliva Samples Spiked with MTX

Analyte	Nominal concentration (µg/ml)	Intra-day RSD%; n=5^a^	Inter-day RSD%; n=5^b^	Accuracy (RE%)^c^	Recoveryd (%)
MTX	25	5.44	7.04	4.54	104.54
	50	7.05	6.19	-2.15	97.85
	100	3.45	3.50	-0.04	99.96

^a^, Number of replicates; ^b^, Number of days; ^c^, RE%=100 ; ((Found value-Nominal value)/ Nominal value). dRecovery (%): Found value/Nominal concentration × 100

**Table 2:** Evaluation of Method Stability of MTX in Saliva

Analyte	Concentration added (µg/ml)	Recovery (%RR)	
		Freeze-thaw stability	Room temperature stability
MTX	50	104.46	104.27
	200	100.28	105.28
	700	98.25	94.22

**Table 3:** Investigation of the Interference Effect on MTX Determination Obtained Under D-MSPE-Spectrophotometry in the Presence of 200 ng/ml MTX

Co-spiked drug with MTX	Accuracy (%)
Morphine	7.8
Vancomycin	8
Clonazepam	7
Cyclosporine	0.5
Citrizine	0.5
Atrovastatine	1.1
Acetaminophen	3.2
Diazepam	7

**Table 4:** Comparison of the Two Methods for the Analysis of MTX in the Saliva Samples of Patients Treated with MTX

Real sample #	MTX level in saliva		t-value^b^	F-value
	Given method^a^	Reference method		
1	0.25 ± 0.010 µg/ml	0.19 ± 0.003 µg/ml	1.15	1.84
2	0.40 ± 0.010 µg/ml	0.58 ± 0.003 µg/ml	-1.88	3

^a^,The found values are average of three determinations ± standard deviation; ^b^, Critical t-value and F-value at %95 confidence level are 4.30 and 19.00. Experimental condition for reference method; Mobile phase, phosphate buffer (pH = 3.6):acetonitrile (89:11, v/v); flow rate, 1 mL/min; λ, 375 nm.

## Discussion

*Application of D-MSPE procedure to real sample*

To evaluate the application of the present method, two saliva samples were taken from patients under MTX therapy and then analyzed by the optimum D-µ-SPE condition. Table 4 shows the patient plasma MTX levels for both samples. The accuracy of the method was confirmed by HPLC measurements. Comparing the results obtained using the current and the reference methods by statistical analysis of the using Student’s t-test and variance ratio F-test showed no statistically significant difference between the performance of the two methods regarding the accuracy and precision, respectively.

The performance of the newly developed D-µ-SPE/spectrophotometric method is comparable with other techniques from the viewpoints of a straightforward technique, LLOQ, apparatus, and validation tests (Albertioni et al., 1995; Hirai et al., 1997; Emara et al., 1998; Turci et al., 2000; Kuo et al., 2006; Chen and Zhang, 2008; Moghbel et al., 2010; Jouyban et al., 2011; Begas et al., 2013; Rodin et al., 2013; Wang et al., 2014; Wei et al., 2014; Wu et al., 2015; Fabrizi et al., 2016; Asadian et al., 2017). For a number of MTX determination LC methods cited in other reports, sophisticated detection systems such as MS or fluorescence were used which are not available in routine bioanalytical laboratories. The present method provided comparable LLOQ (0.01 µg/ml) in comparison with such sophisticated detectors. In addition, the sample preparation step in most of the methods are performed by tedious and time-consuming SPE or LLE techniques. The Present D-µ-SPE method is an improved form of extraction in terms of sensitivity, rapidity, and easiness facility to fix the limitation of both LLE and SPE methods to a great extent.

In conclusion, a comparison of the proposed D-µ-SPE method with other methods shows that D-µ-SPE is a competitive spectrophotometric approach for MTX determination. The consumption of organic solvents in this method is much lower than that in LLE method. The results demonstrate the effectiveness of the D-µ-SPE in quantitatively extracting and pre-treating MTX without tedious pre-treatment procedure and expensive instrumentation. The graphene-based adsorbent was easily prepared and can be well dispersed in the saliva matrix separated from the medium by a centrifuge. This technique is very fast, because the extraction and desorption process take less time (less than 10 min). D-µ-SPE is very simple, low cost, and suitable for clinical operations, which could greatly shorten the sample treatment time. This method is precise and accurate with low quantification limit. The validation criteria meet the FDA requirements very well. This simple method can be applied for the determination of MTX in patients saliva in clinics and laboratories. 
